# Supplementary material for: How did the beginnings of the global COVID-19 pandemic affect mental well-being?
Source: PLoS One. 2023 Jan 20;18(1):e0279753. doi: 10.1371/journal.pone.0279753 (PMC9857989; doi:10.1371/journal.pone.0279753)
Supplement: S1 Table — (PDF) [file pone.0279753.s001.pdf]

S1 Table. Comparison complete cs. incomplete responders

	χ^2	*df*	*p*
Sex	2.38	2	.304
Education	2.04	6	.916
Residence	1.24	4	.871
Essential workers	2.77		.256
High-risk group	3.77	2	.152

[a]due to negative covariances, variance was fixed at zero but included in the modes
