# Supplementary material for: How did the beginnings of the global COVID-19 pandemic affect mental well-being?
Source: PLoS One. 2023 Jan 20;18(1):e0279753. doi: 10.1371/journal.pone.0279753 (PMC9857989; doi:10.1371/journal.pone.0279753)
Supplement: S2 Table — (PDF) [file pone.0279753.s002.pdf]

S2 Table. Correlations between all measured personality facets

	Time 1						Time 5					
	E	A	C	N	O	IPO	E	A	C	N	O	IPO
**Time 1**												
E	1											
A	.29**	1										
C	.3**	.05	1									
N	-0.13	-.27**	-.14*	1.00								
O	.21**	.16*	.16*	.04	1.00							
IPO	-.08	-.34**	-.18**	.37**	-.09	1.00						
**Time 5**												
E	.84**	.31**	.27**	-.24**	.13	-.13	1.00					
A	.26**	.79**	.10	-.24**	.11	-.26**	.36**	1.00				
C	.3**	.11	.71**	-.19**	.14*	-.14*	.37**	.18*	1.00			
N	-.13	-.19**	-.19**	.77**	.03	.33**	-.22**	-.25**	-.23**	1.00		
O	.21	.22**	.10	-.07	.76**	-.11	.26**	.15*	.23**	-.01	1.00	
IPO	-.10	-.28**	-.13	.35**	-.15*	.76**	-.18**	-.28**	-.19**	.42**	-.18**	1.00

[a]due to negative covariances, variance was fixed at zero but included in the modes
