# Supplementary material for: How did the beginnings of the global COVID-19 pandemic affect mental well-being?
Source: PLoS One. 2023 Jan 20;18(1):e0279753. doi: 10.1371/journal.pone.0279753 (PMC9857989; doi:10.1371/journal.pone.0279753)
Supplement: S3 Table — (PDF) [file pone.0279753.s003.pdf]

S3 Table. Means and Standard deviations of SWB

	T1		T2		T3		T4		T5	
SWB	*M*	*SD*	*M*	*SD*	*M*	*SD*	*M*	*SD*	*M*	*SD*
Life Satisfaction	67.97	20.39	67.09	31.79	67.42	23.66	68.7	22.85	71.93	20.5
Stress	37.66	26.5	33.07	27.12	32.68	27.22	33.64	27.21	39.89	27.29
Loneliness			30.55	29.73	29.14	30.14	28.01	29.03	25.1	27.91
Psychological Strain	43.05	25.64	40.83	26.99	38.61	27.04	38.37	27.58	44.17	27.3

ative covariances, variance was fixed at zero but included in the modes
