# Supplementary material for: How did the beginnings of the global COVID-19 pandemic affect mental well-being?
Source: PLoS One. 2023 Jan 20;18(1):e0279753. doi: 10.1371/journal.pone.0279753 (PMC9857989; doi:10.1371/journal.pone.0279753)
Supplement: S4 Table — (PDF) [file pone.0279753.s004.pdf]

S4 Table. Correlations of SWB facets over time

	LS_T1	*LS_T2*	*LS_T3*	*LS_T4*	*LS_T5*	*S_T1*	*S_T2*	*S_T3*	*S_T4*	*S_T5*	*PS_T1*	*PS_T2*	*PS_T3*	*PS_T4*	*PS_T5*	*L_T2*	*L_T3*	*L_T4*	*L_T5*
LS_T1		.64**	.56**	.50**	.51**	-.28**	-.31**	-.29**	-.22**	-.23**	-.40**	-.32**	-.35**	-.31**	-.28**	-.30**	-.29**	-.29**	-.35**
LS_T2	.64**		.68**	.69**	.62**	-.29**	-.33**	-.35**	-.33**	-.14	-.28**	-.37**	-.44**	-.31**	-.24**	-.39**	-.33**	-.34**	-.28**
LS_T3	.56**	.68**		.65**	.53**	-.19*	-.21**	-.26**	-.25**	-.19*	-.19**	-.25**	-.36**	-.29**	-.31**	-.32**	-.33**	-.27**	-.26**
LS_T4	.50**	.69**	.65**		.60**	-.25**	-.23**	-.29**	-.32**	-.15*	-.27**	-.21**	-.31**	-.38**	-.25**	-.25**	-.26**	-.31**	-.21**
LS_T5	.51**	.62**	.53**	.60**		-.22**	-.20**	-.29**	-.29**	-.30**	-.27**	-.30**	-.32**	-.35**	-.42**	-.21**	-.21**	-.30**	-.37**
S_T1	-.28**	-.29**	-.19*	-.25**	-.22**		.60**	.48**	.52**	.30**	.73**	.51**	.40**	.40**	.30**	.01	.09	.09	.22**
S_T2	-.31**	-.33**	-.21**	-.23**	-.20**	.60**		.55**	.50**	.40**	.42**	.70**	.46**	.39**	.38**	.12	.05	.09	.16*
S_T3	-.29**	-.35**	-.26**	-.29**	-.29**	.48**	.55**		.61**	.39**	.38**	.44**	.79**	.52**	.35**	.08	.25**	.20**	.23**
S_T4	-.22**	-.33**	-.25**	-.32**	-.29**	.52**	.50**	.61**		.37**	.35**	.46**	.56**	.77**	.37**	.09	.15*	.29**	.20**
S_T5	-.23**	-.14	-.19*	-.15*	-.30**	.30**	.40**	.39**	.37**		.18*	.34**	.34**	.37**	.82**	.12	.16*	.15*	.31**
PS_T1	-.40**	-.28**	-.19**	-.27**	-.27**	.73**	.42**	.38**	.35**	.18*		.54**	.43**	.46**	.24**	.06	.18*	.14	.21**
PS_T2	-.32**	-.37**	-.25**	-.21**	-.30**	.51**	.70**	.44**	.46**	.34**	.54**		.52**	.49**	.42**	.11	.03	.11	.16*
PS_T3	-.35**	-.44**	-.36**	-.31**	-.32**	.40**	.46**	.79**	.56**	.34**	.43**	.52**		.59**	.36**	.15	.35**	.30**	.19**
PS_T4	-.31**	-.31**	-.29**	-.38**	-.35**	.40**	.39**	.52**	.77**	.37**	.46**	.49**	.59**		.47**	.15*	.21**	.37**	.32**
PS_T5	-.28**	-.24**	-.31**	-.25**	-.42**	.30**	.38**	.35**	.37**	.82**	.24**	.42**	.36**	.47**		.19*	.24**	.27**	.37**
L_T2	-.30**	-.39**	-.32**	-.25**	-.21**	.01	.12	.08	.09	.12	.06	.11	.15	.15*	.19*		.51**	.53**	.34**
L_T3	-.29**	-.33**	-.33**	-.26**	-.21**	.09	.05	.25**	.15*	.16*	.18*	.03	.35**	.21**	.24**	.51**		.59**	.40**
L_T4	-.29**	-.34**	-.27**	-.31**	-.30**	.09	.09	.20**	.29**	.15*	.14	.11	.30**	.37**	.27**	.53**	.59**		.44**
L_T5	-.35**	-.28**	-.26**	-.21**	-.37**	.22**	.16*	.23**	.20**	.31**	.21**	.16*	.19**	.32**	.37**	.34**	.40**	.44**	
