# Supplementary material for: How did the beginnings of the global COVID-19 pandemic affect mental well-being?
Source: PLoS One. 2023 Jan 20;18(1):e0279753. doi: 10.1371/journal.pone.0279753 (PMC9857989; doi:10.1371/journal.pone.0279753)
Supplement: S5 Table — (PDF) [file pone.0279753.s005.pdf]

S5 Table. Summary of model fit statistics for the conditional LGCM including risk group, systemic relevance, sex and age

	N	χ^2 (df)	*p*	CFI	RMSEA	SRMR
**Group differences**						
Life Satisfaction	175	31.49 (20)	.049	.96	.057	.047
Stress	175	29.34 (18)	.044	.954	.06	.04
Psychological Strain	175	34.53 (18)	.011	.932	.072	.039
Loneliness	175	5.85 (8)	.664	1.0	.000	.022
