# Supplementary material for: How did the beginnings of the global COVID-19 pandemic affect mental well-being?
Source: PLoS One. 2023 Jan 20;18(1):e0279753. doi: 10.1371/journal.pone.0279753 (PMC9857989; doi:10.1371/journal.pone.0279753)
Supplement: S6 Table — (PDF) [file pone.0279753.s006.pdf]

S6 Table. Estimations of intercept and slope, effects of group differences, sex and age

Model		x̄	s	gender	High-risk group	Essential workers	age	R^2
Life Satisfaction	i	68.15**	258.35**	0.63	2.75	-3.36	0.1	.02
	s	8.62	1.89	-5.07*	-0.97	-0.12	-0.06	.77
	q	1.58	-0.08	-1.14	-0.3	0.26	-0.01	a
Stress	i	41.88**	452.2**	-10.97*	5.91	-2.00	-0.01	.07
	s1	5.72	430.32*	11.9	-5.46	-8.36	-0.25	.1
	s2	-4.23	26.61*	-1.11	0.71	0.43	0.13*	.11
Psychological Strain	i	41.06**	418.73*	-8.03	4.77	-1.25	0.11	.04
	s1	8.86	257.91	5.51	-4.26	-5.76	-0.35	.1
	s2	-1.49	27.57*	-0.63	-0.07	-0.32	0.09	.06
Loneliness	i	10.68	392.45**	-0.75	10.63	7.92	0.04	.08
	s	27.25*	522.67	-7.77	-18.17*	-5.50	-0.14	.11
	q	-3.66	33.7	1.47	3.89	0.34	-0.01	.09

[a]due to negative covariances, variance was fixed at zero but included in the modes
