# Supplementary figures and images for: How did the beginnings of the global COVID-19 pandemic affect mental well-being?
Source: PLoS One. 2023 Jan 20;18(1):e0279753. doi: 10.1371/journal.pone.0279753 (PMC9857989; doi:10.1371/journal.pone.0279753)

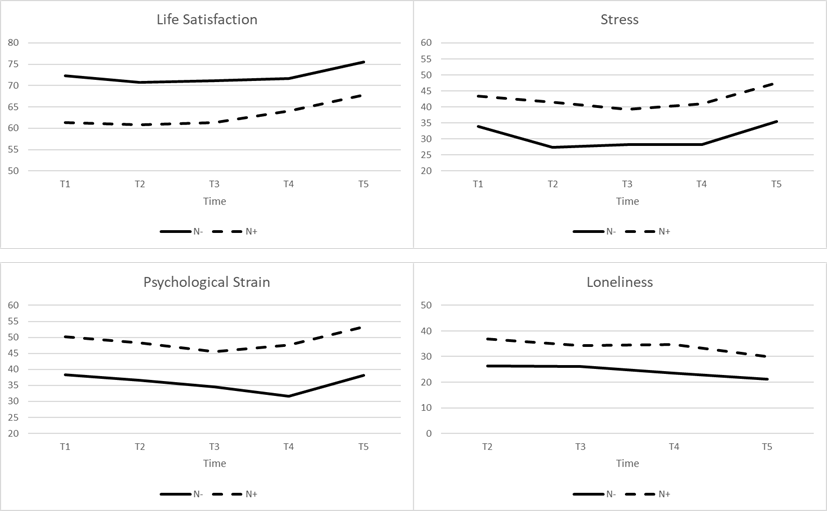

Supplement: S1 Fig — (TIF) [file pone.0279753.s007.tif]

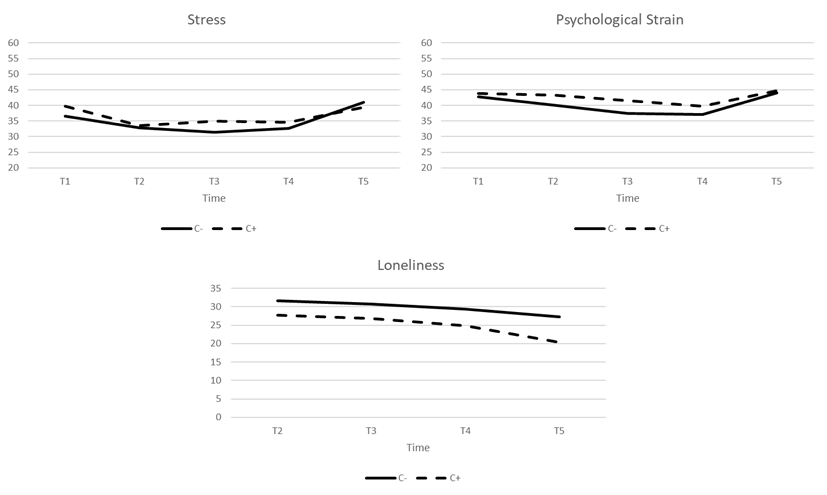

Supplement: S2 Fig — (TIF) [file pone.0279753.s008.tif]

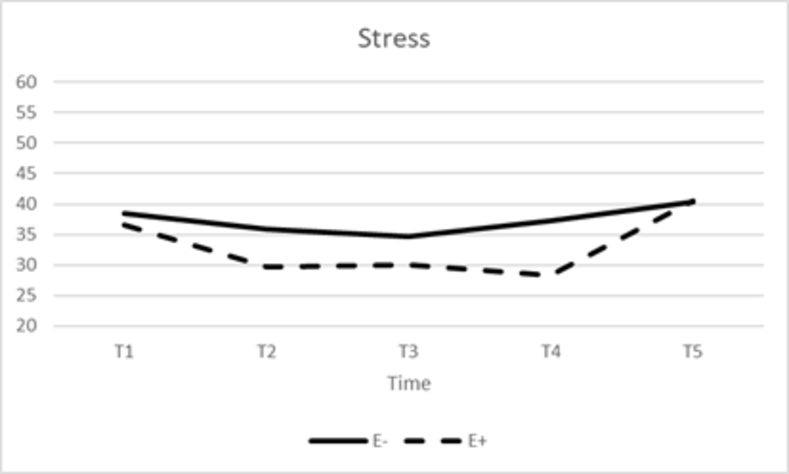

Supplement: S3 Fig — (TIF) [file pone.0279753.s009.tif]

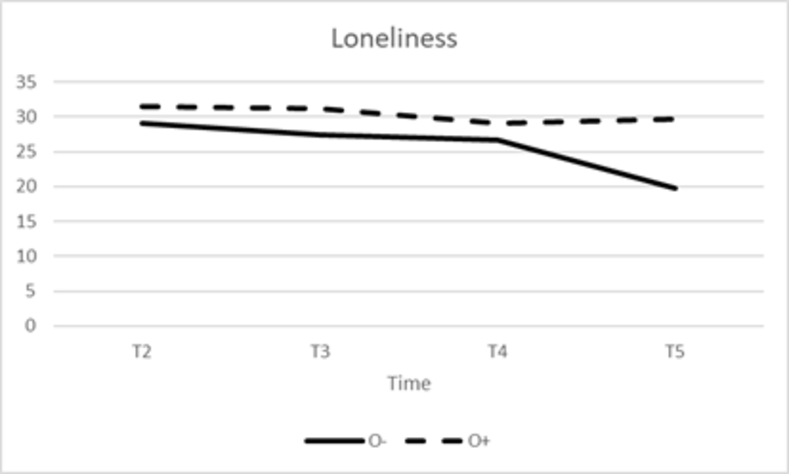

Supplement: S4 Fig — (TIF) [file pone.0279753.s010.tif]

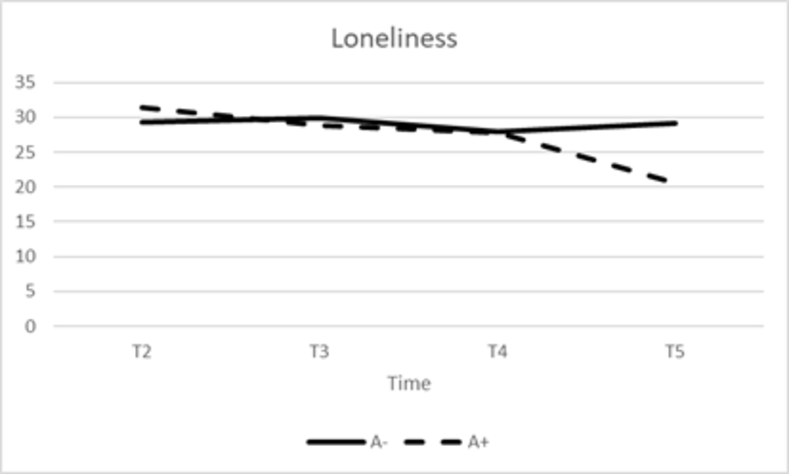

Supplement: S5 Fig — (TIF) [file pone.0279753.s011.tif]
