# Supplementary material for: How did the beginnings of the global COVID-19 pandemic affect mental well-being?
Source: PLoS One. 2023 Jan 20;18(1):e0279753. doi: 10.1371/journal.pone.0279753 (PMC9857989; doi:10.1371/journal.pone.0279753)
Supplement: Attachment — Submitted filename: Response to Reviewers.docx [file pone.0279753.s012.docx]

Dear Dr. Vega-Muñoz,

Thank you very much for your comments on our manuscript titled “How did the beginnings of the global COVID-19 pandemic affect mental well-being?” (PONE-D-22-21116). We are also grateful for the reviewers’ comments and have now revised the paper in accordance with this advice. The revisions we made are highlighted in the file labelled 'Revised Manuscript with Track Changes'.

Below you can find the requested point-by-point responses in which we address all the concerns raised by you and the reviewers. For reasons of simplicity, we answered comments addressing the same issues together. We hope that we were able to reconcile all the concerns in an acceptable way, and we are looking forward to hearing from you again.

Yours sincerely,

XXX (names omitted due to blind review)

**Reviewer #1:** I like this study, but the discussion suffers from a neglect of a highly relevant recent study (Getzmann et al., COVID-19 Pandemic and Personality: Agreeable People Are More Stressed by the Feeling of Missing, Int. J. Environ. Res. Public Health 2021, 18(20), 10759). This longitudinal study from Germany compared - in the same sample- stress levels that were obtained pre-Corona to post-Corona stress levels and related these changes to personality (Big 5) that was also measured pre-Corona. The results partly confirm the findings of the present study but also reported patterns that clearly diverge from the present findings (esp. wth respect to the relation of agreeableness and loneliness). I find it mandatory to consider this study in the discussion of the present paper.

R#1 A: Thank you for drawing our attention to this study. We found it interesting and helpful, especially because of the analysed relationship between changes in stress and the feeling of missing out. The moderation found in this study, should – in our option –also be considered. Therefore, we included the study in our discussion (future perspective paragraph) instead of the introduction for two reasons. Including this study in our introduction creates the impression that this study could have influenced our pre-registered hypotheses, thus portraying our research process in a potentially misleading way. Second, the measures used by Getzmann and colleagues differed substantially from our measures (e.g., their pre-covid measure spanned 22.5 months, resulting in very different pre-post-test intervals) which limits the transferability to our findings. Nonetheless, we agree that it is important to report the findings of this study which can inspire future research projects. Therefore, we added the following section to our manuscript:

*“**In addition**, the relation between different mental well-being measures (e.g., Stress, Loneliness) and SWB facets (e.g., Life Satisfaction) as well as the moderation of this relationship through personality should also be investigated. Getzmann and colleagues (66) found a moderating effect of Agreeableness on the relation between Feeling of Missing and changes in* *stress* in terms of higher agreeableness leading to a stronger association of feeling of missing and stress, *measured both pre-COVID-19 and after the lockdown. It is therefore possible, that other personality facets, which did not seem relevant in our study**, exert their effect through moderation processes with well-being and related stress measures.”*

**Reviewer #2:** This study is an investigation of mental health changes in response to the COVID-19 crisis. The sample, though relatively small, is followed up to 5 times over the first months of the pandemic in Germany. The manuscript is well-written and the authors clearly specify their research questions. However, there are a few aspects of the study that undermined my enthusiasm.

*Majors:*

1. Your terminology and definition of concepts. The authors refer to SWB (subjective well-being), but what they measure are different components of mental or psychological health. Per theory, SWB has three components: life satisfaction (LS), positive affect (PA), and negative affect (NA) (Andrews & Withey, 1976). Individuals are said to have high SWB if they experience LS and frequent PA (e.g., joy, optimism) and infrequent NA (e.g., sadness, anger). The authors only measure LS, and include measures of psychological strain, stress and loneliness, which in my view are constructs that are correlated but differ from subjective well-being. The authors noted this a limitation of their study (p. 37), but I would rather recommend them to revise their language and terminology in the manuscript and refer to mental health or mental well-being (as similarly done in the Measure section, p. 16).

R#2 A1: We fully agree that we did not capture all aspects of SWB in our measurement. Although SWB is measured in many studies by the main indicator of life satisfaction and we thus chose this designation, we understand the objection. Therefore, we revised our language and terminology and now refer to mental well-being instead. However, the limitations about SWB addressed in the manuscript remain, because we find it worth noting that other aspects such as fear, anxiety, and happiness should also be taken into account.

2. Personally, I share the urgency to understand the impact of the COVID-19 pandemic on mental health, across different at-risk groups (for example, essential workers). However, in the introduction, a better job could be done to clearly state what is the unique contribution of this work. Moreover, one missing piece in the published literature is to understand long-term consequences of the pandemic. Have the authors considered to pair their data with other public available COVID-19 surveys? For example, SHARE (<http://www.share-project.org/home0.html>) includes similar mental health measures from a subsample from Germany, which might make possible to compare COVID-19 consequences on mental well-being, short-term and over 1 year, in the same country.

R#2 A2: In order to illustrate the unique contribution of our work to COVID-19 related research more clearly, we have added the following section:

*“In contrast to other studies, we placed a strong emphasis on the analysis of the trajectory of mental well-being by administering questionnaires at short intervals of weekly surveys during the lockdown. This approach allows for a closer inspection of changes throughout this phase of the pandemic.”*

We fully share your opinion that the combination of datasets and multiple-lab approaches benefit psychological research. Upon closer inspection of the SHARE data as well as further datasets (such as SOEP), we noticed too many substantial differences that render direct comparisons difficult without straying too far from the original research idea that was pre-registered. To further emphasize the importance of replication and combination with other data sets, however, we have included the following paragraph:

*“**Especially because of the global nature of the COVID-19 pandemic, it is important to replicate results in other samples and in other countries. In addition, since the pandemic has been ongoing for more than two years now, the impact of the first lockdown (or further lockdowns) should also be analysed in longer follow-up designs. In this way, long-term effects of the lockdowns can be investigated and action recommendations with regard to potential future pandemic developments can be derived. It is conceivable that influences of personality facets and the group differences we found in our study will continue to be important for mental health, coping and dealing with the pandemic. Again, further analyses of various panel data may be helpful in this regard. This view is supported by the fact that some longitudinal surveys (e.g., SOEP, SHARE) have included additional interviews that specifically address the COVID-19 pandemic and related well-being.”*

3. In term of analytical approach, the models seem adequately specified. However, for the model including personality as predictor of mental health changes, I would recommend to each personality measure in separate model and have the model with all traits entered together as a follow-up analysis.

R#2 A3: Thank you for this suggestion. To test this approach, we set up each model including only one personality measure. Those resulted in a very similar pattern, even though some numerical values were a little bit higher (which could have been expected given the fact that the shared variance between personality facets is not represented in this approach). Nevertheless, including personality measures separately did not lead to substantial changes in the results and have therefore been omitted from the manuscript. Furthermore, since the separate models do not account for the variance shared between each personality measure, the separately including procedure may yield somewhat biased estimates. Therefore, we decided to hold on to the reported models including all five personality measures as well as their intercorrelations simultaneously. To justify this decision to the readers we added the following to our analytic strategy section:

*“To analyse the combined effect of personality, all personality factors (Big Five and Personality Organisation) were modelled simultaneously, while controlling for age. This approach allows us to estimate the effect of each personality factor while controlling for the effect of all other factors.”*

4. In the discussion, please consider that personality might change as well in response to the COVID-19, as supported by recent evidence published in Plos One (Sutin et al. 2020, DOI: 10.1371/journal.pone.0237056; Sutin et al. in press).

R#2 A4: Thank you for raising this important issue. We added the study from Sutin and colleagues (2020) to our discussion. Additionally, we analysed differences in our measured personality facets from the first survey and the fifth. In our sample, significant differences were found for Neuroticism, indicating lower values after the lockdown (M_1st_ = 2.88, SD_1st_ = 0.8; M_5th_ = 2.74, SD_5th_ = 0.84, *t(209)* = 3.31, *p* = .001, *d* = .234). Even if the effect we found in our sample is very small, our data support the interpretation by Sutin and colleagues in the sense that people seem to attribute their worries to some degree on the COVID-19 pandemic rather than on their own personality now. To investigate if those changes lead to different results in our LGCM, we further explored the model estimations including the Neuroticism-score from the last survey period. Since this adjustment did not change our results, we added the following:

*“**Additionally, it should be considered that personality might change in response to the COVID-19 pandemic. Along these lines, Sutin and colleagues (65**) report a decrease in Neuroticism scores during the first six weeks of the pandemic.* *Their interpretation of this change was that people attribute their feelings of anxiety and distress more on the pandemic and less on their own personality. In our study, we also found a significant decrease in Neuroticism between the first and the last survey. Nevertheless, the LGCM results remained unchanged when Neuroticism scores from the last survey were included.”*

5. Further, under study limitation (or direction for future studies), more stress should be placed on the need to replicate study results in future work and the use of longer follow-up (related to point 2).

R#2 A5: see R#2 A2

*Minors:*

6. Abstract, the authors do not include any information on sample size, study design nor sample characteristics (for example, age).

R#2 A6: We added information concerning our analysed sample as well as a short summary of the methodology used.

*“[…] Overall, 272 adults (M_age_= 36.94, SD_age_= 16.46; 68.62 % female, 23.45 % male, 0.69 % non-binary) took part in our study with four weekly surveys during the lockdown as well as a follow-up one month after restrictions were lifted. To analyse the development of mental well-being during and shortly after the first lockdown in Germany latent growth curve models (LGCM) were calculated. The considered facets of well-being differ by their trajectory. […]”*

**Reviewer #3:** I think it is a very interesting research, of great relevance to the context of the Covid-19 pandemic, and it is very well written. However, I recommend in the abstract to briefly indicate the methodology, and in the article in general, to improve the concordance between the concepts presented and what was measured, because although it talks about subjective well-being, which in the same introduction, it is mentioned that it is made up of the components life satisfaction, positive affect and negative affect, not all of these are measured.

R#3 A: Thank you for your comment on the abstract. We now briefly indicate the methodology in our abstract (see Reviewer #2, comment 6). We agree that we did not have a well-suited name for our measured well-being facets. Therefore, we followed the suggestion of Reviewer #2 to revise our language and terminology in the manuscript. We now define our measured components as mental well-being.
